# Root morphology, nitrogen metabolism and amino acid metabolism in soybean under low phosphorus stress

**DOI:** 10.1038/s41598-024-79876-0

**Published:** 2024-11-19

**Authors:** Meiling Liu, Mingzhe Zhao, Guang Yang, Mingze Sun, Ahui Yang, Chang Sun, Hongyu Zhao, Xue Ao

**Affiliations:** College of Agronomy, Shenyang Agricultural University, Shenyang, 110866 Liaoning China

**Keywords:** Soybean, Roots, Phosphorus deficiency, Metabolic pathway, Physiology, Plant sciences

## Abstract

Phosphorus deficiency is a major influence on growth and development of soybean. Therefore, improving phosphorus utilization efficiency in soybean is a research priority for the soybean community. In this experiment, Liaodou 13 (high phosphorus utilization: HPE) and Tiefeng 3 (low phosphorus utilization: LPE) were used as test varieties. We investigated changes in root morphology, amino acid content, and content of key substances of the nitrogen metabolic pathway with normal phosphorus (0.5 mM) and low phosphorus (0.005 mM) treatments. The results showed that the root length, root surface area and number of lateral roots of HPE roots were higher than those of LPE roots under normal and low phosphorus conditions. The contents of different types of amino acids showed different trends in two varieties. The HPE showed small change in the content of total hydrolyzed amino acids under the low phosphorus condition when compared to the normal phosphorus treatment by a 6.67% decrease, on the contrary LPE showed a drastic decrease by 20.36%. However, HPE exhibited similar decreasing trends in the contents of hydrolyzed and free aspartic acid with the low phosphorus treatment. Moreover, the contents of free histidine and valine in LPE were significantly increased by 657.84% and 149.29% respectively, in contrast to significant decreases in HPE. In aspects of major nutrient elements, the contents of phosphorus, total nitrogen and ammonia nitrogen in both HPE and LPE varieties decreased to dramatic levels. However, the nitrate nitrogen content significantly increased 78.51% for HPE and 65.12% for LPE. Compare to the normal condition, the GOGAT activity in HPE decreased by 5.18% but increased by 33.10% in LPE. Compare to the normal condition, the GS activity in HPE increased by 7.26% but decreased by 21.72% in LPE under phosphorus deficiency. In summary, the phosphorus-efficient soybean variety HPE exhibited superior tolerance to low phosphorus deficiency through advantageous root morphology, phosphorus uptake and transfer capability, and balanced amino acid metabolism and nitrogen metabolism pathways.

**Supplementary Information:**

The online version contains supplementary material available at 10.1038/s41598-024-79876-0.

## Introduction

Phosphorus is an essential element required for the synthesis of nucleotides, proteins and phospholipids, which plays a vital role in supporting plant growth and development^1^. However, phosphate rock is expected to be depleted within centuries as a major source of phosphorus^2^. Hypomineralization reduces phosphorus uptake by plants and affects plant growth and productivity^3^. Although the application of chemical fertilizers can alleviate the problem of phosphorus deficiency to a certain extent, there is also the risk of resource waste and environmental pollution^4^. Therefore, breeding crop varieties with high tolerance to phosphorus deficiency has become an efficient and environmentally friendly strategy to achieve sustainable agricultural production. Recent comprehensive genetic studies of plant nutrition have demonstrated the existence of various traits related to phosphorus uptake and utilization among different crops, including soybean^5^, maize^6^, and rice^7^. In addition, these characteristics have been observed between different varieties of the same species. Mechanistic studies investigating differences in phosphorus efficiency are usually carried out using contrasting crop varieties.

Previous studies related to low phosphorus mainly focused on root conformation changes and genetic foundation for improving crop phosphorus tolerance, however on the physiological level interactions between physiological and nitrogen metabolism are much less studied. Higher plants have evolved a range of adaptive mechanisms to cope with phosphorus deficiency stress, involving changes in root conformation, starch biosynthesis, sugar metabolism, and secreted organic matter biosynthesis. These physiological and biochemical changes enhance the uptake and utilization of phosphorus by the plant for better development and growth under limited phosphorus conditions^8^. In addition, the symbiotic relationship between plant roots and rhizosphere microorganisms (e.g., mycorrhizal fungi and rhizobacteria) plays an important role in maintaining phosphorus homeostasis^9^. Nitrogen metabolism is one of the most basic metabolism networks in plants. Plant nitrogen metabolism converts inorganic nitrogen to organic nitrogen through the processes of nitrate reduction, ammonia nitrogen assimilation, and amino acid synthesis with key enzymes such as nitrate reductase (NR), glutamine synthetase (GS), and glutamate synthetase (GOGAT)^10,11^. In higher plants most majority of ammonia assimilation is through the GS/GOGAT (glutamine synthetase/glutamate synthetase) cycle, which produces glutamine and glutamate precursors for the synthesis of derivative amino acids. The ammonia sources in legume roots for assimilation include ammonia absorbed from the soil and produced by the nitrate reduction. Downregulation of the GS/GOGAT cycle leads to reduced levels of nitrogen metabolism. Phosphorus deficiency decreases the activities of key enzymes involved in nitrogen metabolism^12^. Previous studies claimed that prolonged low phosphorus significantly downregulates the primary GS/GOGAT cycle in nitrogen metabolism^13^. Also there were reports that low phosphorus stress promoted the activities of key enzymes of nitrogen metabolism in peanut (e.g., NR, glutamate dehydrogenase (GDH) and GS)^14^.

The adaptive response of soybean (*Glycine max*L.) to phosphorus (P) deficiency is complex and is influenced by several genetic factors^15,16^. The regulatory mechanisms affecting phosphorus utilization in soybean involve complex changes in multiple tissues and levels, including phenotypic, physiological, and biochemical levels of metabolism, especially those related to amino acid metabolism. However, a comprehensive understanding of these changes in physiological and metabolic levels is lacking and further in-depth studies are needed. Since soybean varieties with different phosphorus efficiencies previously showed differences in glycolysis and amino acid-related pathways on the molecular level^17^, we focused on the characterization of differential responses in morphology, amino and nitrogen metabolism in soybean roots. We investigated the effects of low phosphorus stress on the morphological changes of soybean roots, the content of key substances of nitrogen metabolism pathway and the content of various amino acids. It provides a theoretical basis for stress-resistant cultivation of soybean by providing insight into the differences in physiological effects of low phosphorus stress on soybean seedlings.

## Materials and methods

### Plant material

Two soybean cultivars were selected for this experiment: high P efficiency Liaodou 13 (HPE) and low P efficiency Tiefeng 3 (LPE)^17^. The test varieties Tiefeng 3 and Liaodou 13 were provided by Shenyang Agricultural University. The experiment was conducted at the experimental station of Shenyang Agricultural University in a randomized block design with three replications using the sand culture method. PVC pots with an inner diameter of 16 cm, a height of 25 cm and three 0.2 cm holes in the bottom were used as culture containers. In this experiment, KH_2_PO_4_ was used as a source of P at 0.5 mM for normal phosphorus treatment (NP) and 0.005 mM for low phosphorus treatment (LP), and KCl was used as a potassium supplement.

Five seeds were sown in each PVC pot. Every day, 500 ml of distilled water was poured at 8:00 am and 1000 ml of distilled water at 4:00 pm. After germination, three seedlings of uniform growth were retained in each pot and supplemented with 500 mL of half-concentrated nutrient solution and 1,000 mL of distilled water at 8:00 a.m. and 4:00 p.m. daily, respectively. Nutrient solution cultured soybean seedlings for 14 days followed by LP treatment. At day 7 after LP treatment, soybean leaves were taken and snap-frozen in liquid nitrogen for subsequent physiological experiments. Three pots were used as replicates. Total nine plants for each replicate were individually harvested as single plants. Three plants (one plant from each pot) with uniform growth statue were selected for examination and data collection. The total nutrient solution was formulated with the following components: 3.6 mmol·L^−1^ CaSO_4_·2H_2_O, 2 mmol·L^−1^ KNO_3_, 18 umol·L^−1^ FeSO_4_·7H_2_O, 18.9 umol·L^−1^ KCl, 9.3 umol·L^−1^ H_3_BO_3_, 0.9 umol·L^−1^ MnSO_4_·H_2_O, 0.9 umol·L^−1^ ZnSO_4_·7H_2_O, 0.18 umol·L^−1^ CuSO_4_·5H_2_O, 0.18 umol·L^−1^ (NH_4_)_6_Mo_7_O_24_·4H_2_O, 250 umol·L^−1^ MgSO_4_·7H_2_O. The pH of the nutrient solution was adjusted to 6.0^18^.

### Root phenotyping

Roots of soybean seedlings under different phosphorus treatments were carefully washed using distilled water to remove adherent quartz sand, and seedlings were dissected at the cotyledonary nodes. Root length, root surface area and number of root tips were determined in a water bath using a root scanner (Win-RHIZO 2014 software).

The collected root samples were baked in an oven at 105℃ for 30 min. Then, the temperature was lowered to 80℃ and the samples were allowed to dry to a constant weight. Transfer 0.15 g of the ground dried root sample to a digestion tube and added 4 mL of concentrated sulfuric acid. The mixture is maintained at 330℃ for 7 min. After cooling, 2 mL of hydrogen peroxide was added to the sample and then heated again to 330℃ until the mixture became clear. After cooling to room temperature, distilled water was added to the digest to bring the total volume to 50 mL for subsequent analysis. Total phosphate content was determined at 710 nm using a SmarChem automated chemistry analyzer^19,20^.

### Measurement of amino acid content

Measurements of amino acid contents using an Agilent 1260 liquid chromatography system were performed as previously described^21^. To calculate the conversion factor (ConF), the ratio of Asp content (g·kg^−1^) against the total content (g·kg^−1^) of four amino acids (Met, Iso, Lys and Thr which all can be synthesized from Asp) was calculated first and named as AspR. The ratio between AspR under the NP condition (NP-AspR) and the AspR under the LP condition (LP-AspR) was the ConF of a variety. AspR = Asp/(Met + Iso + Lys + Thr); ConF = NP-AspR/ LP-AspR. The logarithm value of a ConF was taken against two and defined as ConF (log2).

### Enzyme activity measurement

All enzyme activities were assayed using commercially available kits and assayed according to the instructions provided by Beijing Solarbio Science & Technology Co., Ltd.

### Statistical analysis

An ANOVA model was used for experimental data analysis. All data were analyzed by two-way ANOVA using SPSS24.0 software. Duncan’s test was used to evaluate differences, among different treatments and *P* < 0.05 indicated that there was a statistically significant difference between treatments. Means and standard deviations were calculated, and the LSD test was used for examination of means. Three replications of each metric for each treatment were used. The graphics were drawn using Origin9.1 software.

## Results

### Differential root growth under low phosphorus stress

**Fig. 1:**
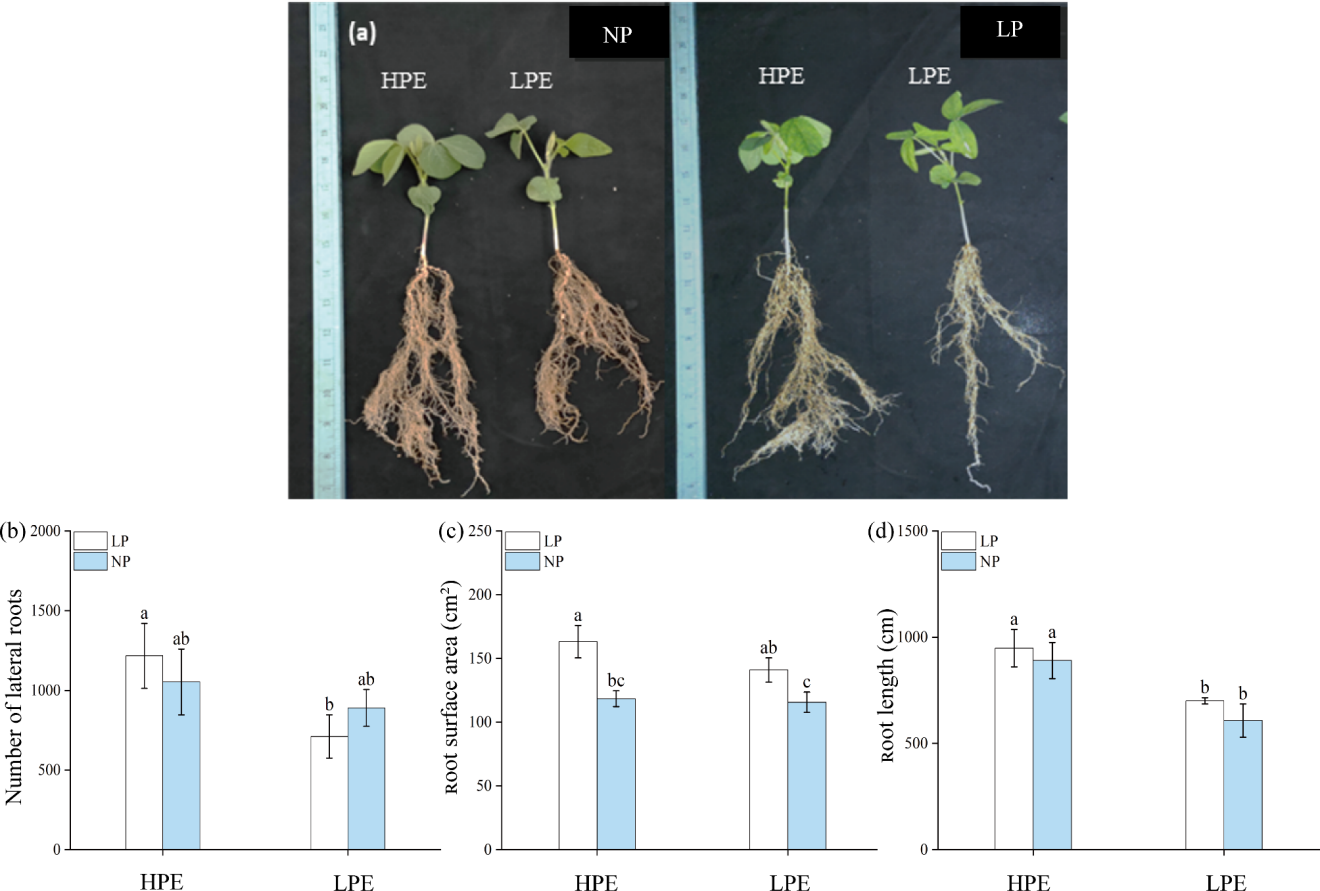
Root distribution of soybean varieties with different phosphorus efficiencies in different phosphorus treaments. Low Phosphorus Treatment: LP; Normal Phosphorus Treatment: NP; High phosphorus efficiency variety (Liaodou 13) and low phosphorus efficiency variety (Tiefeng 3) are labelled as “HPE” and “LPE” respectively. (**a**) Morphology of soybean. (**b**) Number of lateral roots. (**c**) Root surface area. (**d**) Root length. (**e**) Root diameter classification. Columns represent the means ± SDs for three biological replicates. Treatments with the same letter are not significantly different from each other at a p-value level of 0.05.

In LP treatment, root surface area and total root length were slightly increased in HPE and LPE than in NP condition. In NP and LP treatments, the number of lateral roots, root surface area and total root length were higher in HPE than in LPE (Fig. 1a-d). In NP treatment, total root length of HPE was significantly higher by 32.42% than that of LPE (Fig. 1d). Compare to LPE, the number of lateral roots and total root length of HPE significantly increased by 69.80% and 28.20% under phosphorus deficiency (Fig. 1b, d). The differences in lateral root number and total root length in HPE between LP and NP treatments were insignificant (Fig. 1b, d), but root surface area was significantly higher in LP stress than in NP treatment (Fig. 1c). Compared with NP treatment, under LP treatment, the length changes of the fine roots in HPE variety were smaller than in LPE variety. In LPE length of the fine roots reduced by 22.72% and 5.07% in the first and second category respectively (Table 1). These results indicated that the root morphological configuration was differentially promoted by LP stress in soybean varieties with different phosphorus efficiencies. Our HPE variety presented a more advantageous root system.

**Table 1:** Effect of low phosphorus on length of soybean fine roots under different diameter categories.

Variety	Phosphorus treatment	Root category I(0–0.5 mm)	Root category II(0.6–1 mm)	Root length change category I	Root length change category II
HPE	LP	505.85	265.31	−4.02%	−2.90%
	NP	485.51	257.62	-	-
LPE	LP	387.14	247.41	22.72%	5.07%
	NP	500.94	260.62	-	-

Note: Low Phosphorus Treatment: LP; Normal Phosphorus Treatment: NP; High phosphorus efficiency variety (Liaodou 13) and low phosphorus efficiency variety (Tiefeng 3) are labelled as “HPE” and “LPE” respectively. Soybean fine roots were divided into into two categories with different diameters. Root category I: diameter range of 0–0.5 mm; root category II: diameter range of 0.6–1 mm. Columns represent the average root length in unit of centimeters. Root length change=(NP-LP)/NP.

### Changes in hydrolyzed and free amino acid contents

#### Changes in hydrolyzed amino acid content

**Fig. 2:**
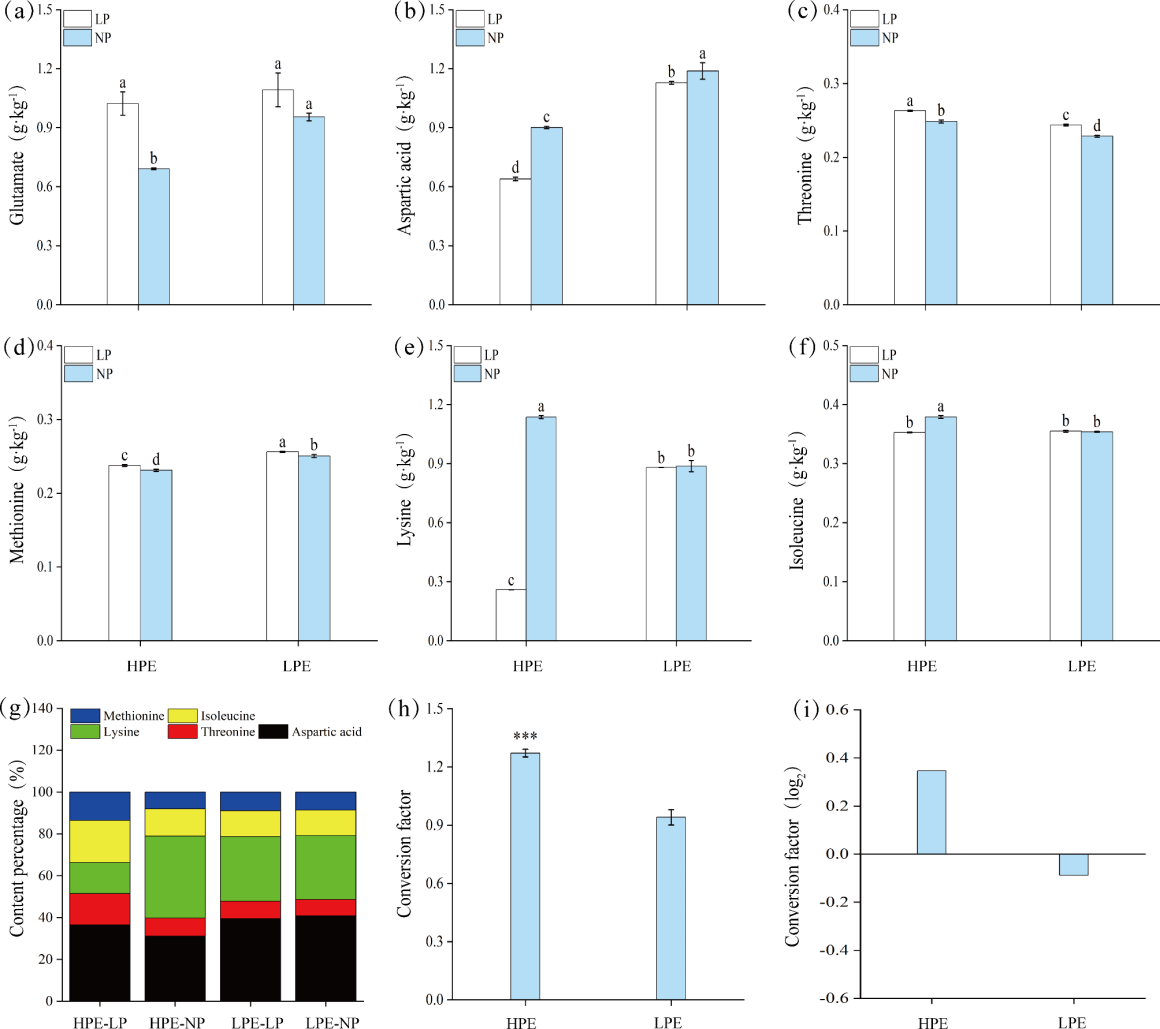
Comparative analysis of six hydrolyzed amino acids, allocation and aspartic acid conversion. Low Phosphorus Treatment: LP; Normal Phosphorus Treatment: NP; High phosphorus efficiency variety (Liaodou 13) and low phosphorus efficiency variety (Tiefeng 3) are labelled as “HPE” and “LPE” respectively. (**a**) Hydrolyzed glutamate. (**b**) Hydrolyzed aspartic acid. (**c**) Hydrolyzed threonine.(**d**) Hydrolyzed methionine.(**e**) Hydrolyzed lysine.(**f**) Hydrolyzed isoleucine. (**g**) Percentage of major hydrolyzed amino acid content. (**h**) Conversion of free aspartic acid. (**i**) Conversion factor(log_2_). Columns represent the means ± SDs for three biological replicates. Treatments with the same letter are not significantly different from each other at a p-value level of 0.05.

Amino acids stimulate cell division and growth of root-end meristematic tissues, increase root length and volume, and improve the efficiency of nutrient uptake. Therefore, amino acid content in roots was determined to further understand the effects of LP stress on the root system. To study the differences between HPE and LPE on the level of amino acid metabolism, we first compared contents of six major hydrolyzed amino acids in two varieties under both phosphrous treatments. We found that in NP treatment contents of hydrolyzed threonine, lysine and isoleucine in HPE were significantly higher than those in LPE (Fig. 2c, e, f), but with relatively lower HPE glutamate, aspartic acid and methionine contents(Figure 2a, b, d). In LP treatment, HPE only showed higher threonine content by 4.00% (Fig. 2c) but dramatic lower contents in hydrolyzed aspartic acid, lysine and isoleucine by 28.89%、77.19% and 7.89% respectively (Fig. 2b, e, f). Also there was no significant difference in contents of hydrolyzed glutamate and isoleucine between HPE and LPE under LP stress (Fig. 2a, f). Then we examined the changes in amino acid contents under LP stress compared to NP treatment. We found that under LP stress HPE and LPE exhibited similar increasing trend in contents of hydrolyzed glutamate, threonine and methionine (Fig. 2a, c, d). Other than that, HPE showed decreasing in contents of aspartic acid, lysine and isoleucine (Fig. 2b, e, f) while LPE only decreased in aspartic acid content (Fig. 2b) with no change in lysine and isoleucine contents (Fig. 2e, f). Last, what deserved to be pointed out was that we observed much higher contribution from hydrolyzed lysine and isoleucine than from other examined amino acids in both HPE and LPE (Fig. 2g), and also that the conversion efficiency of hydrolyzed aspartic acid was significantly higher in HPE than in LPE under LP stress (Fig. 2h, i).

#### Changes in free amino acid content

**Fig. 3:**
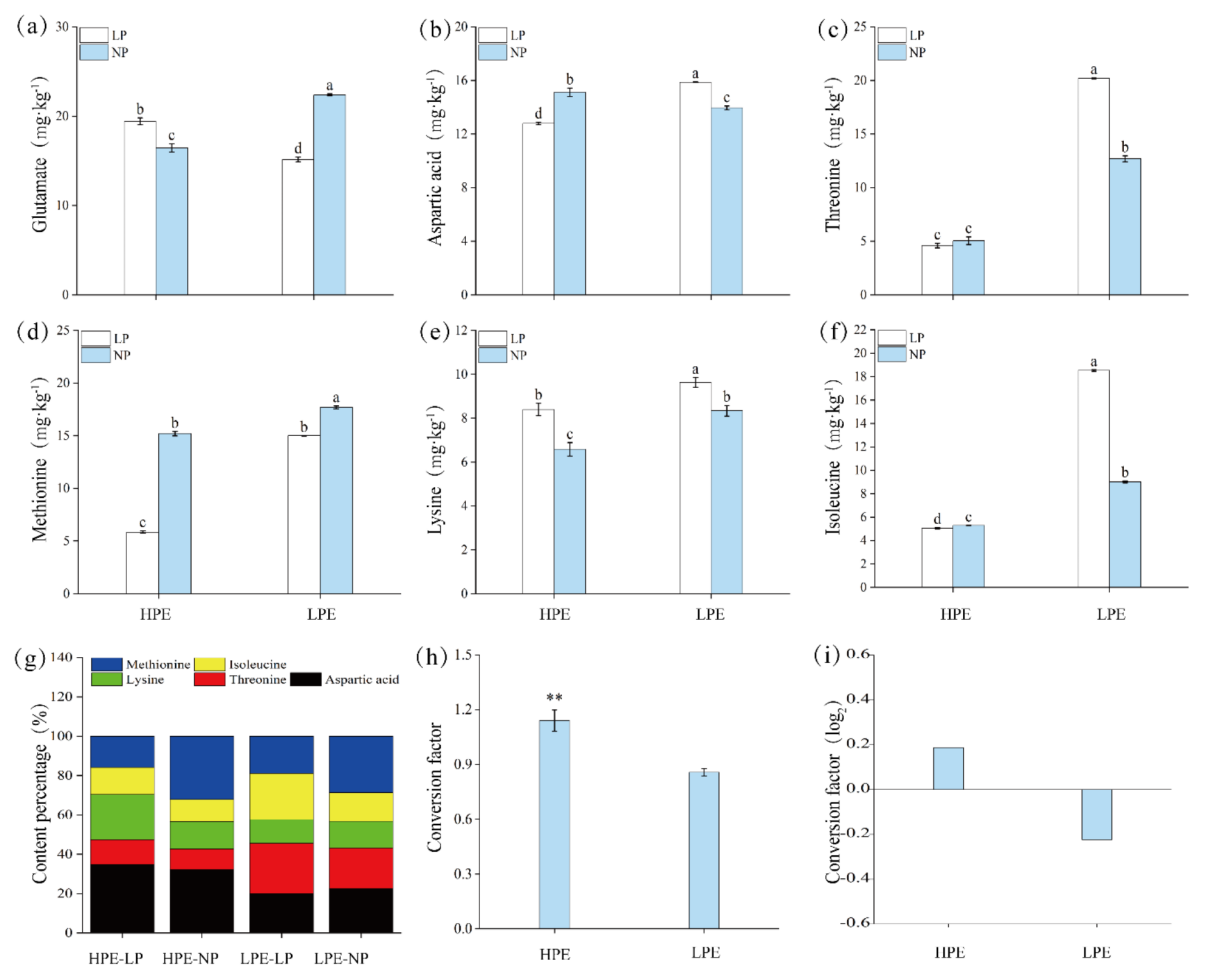
Comparative analysis of six free amino acids, allocation and aspartic acid conversion. Low Phosphorus Treatment: LP; Normal Phosphorus Treatment: NP; High phosphorus efficiency variety (Liaodou 13) and low phosphorus efficiency variety (Tiefeng 3) are labelled as “HPE” and “LPE” respectively. (**a**) Free glutamate. (**b**) Free aspartic acid. (**c**) Free threonine. (**d**) Free methionine. (**e**) Free lysine. (**f**) Free isoleucine. (**g**) Percentage of major free amino acid content. (**h**) Conversion of free aspartic acid. (**i**) Conversion factor (log2). Columns represent the means ± SDs for three biological replicates. Treatments with the same letter are not significantly different from each other at a p-value level of 0.05.

Next, we examined changes in contents of free major amino acids following the same analytical strategy as with hydrolyzed amino acids. We found that in NP treatment HPE only possessed higher content in free aspartic acid than LPE by 8.31% (Fig. 3b), but with significant lower levels of all other five free major amino acids including glutamate, lysine, methionine, threonine and isoleucine (Fig. 3a, c-f). Similarly, HPE still showed lower levels of almost all free major amino acids (Fig. 3b-f) in LP treatment except a higher glutamate content which was increased by 28.17% (Fig. 3a). We also observed different change patterns in free amino acids between HPE and LPE under LP stress. Compared to NP treatment, HPE showed increasing in glutamate and lysine (Fig. 3a, e) but decreasing in aspartic acid, methionine and isoleucine (Fig. 3b, d, f). On the contrary, LPE showed increasing in free aspartic acid, threonine, lysine and isoleucine (Fig. 3b, c, e, f) and decreasing in free glutamate and methionine (Fig. 3a, d). The only same pattern for HPE and LPE was increasing in lysine and decreasing in methionine. And these two varieties exhibited exactly opposite change patterns in free glutamate and aspartic acid the two key amino acids. Under LP stress, the two most contributing amino acids were aspartic acid and threonine and the conversion efficiency of aspartic acid was drastically higher in HPE than in LPE (Fig. 3h, i). In summary, when contents of hydrolyzed and free amino acids were considered together, HPE possessed higher total glutamate than LPE (Figs. 2a and 3a). More importantly, HPE contained lower levels of aspartic acid and higher conversion efficiencies than LPE under LP stress which suggested that the conversion of aspartic acid into other molecules was probably the cause of its lower content level (Figs. 2b and i and 3b and i).

### Changes in the content of major nutrient elements

**Fig. 4:**
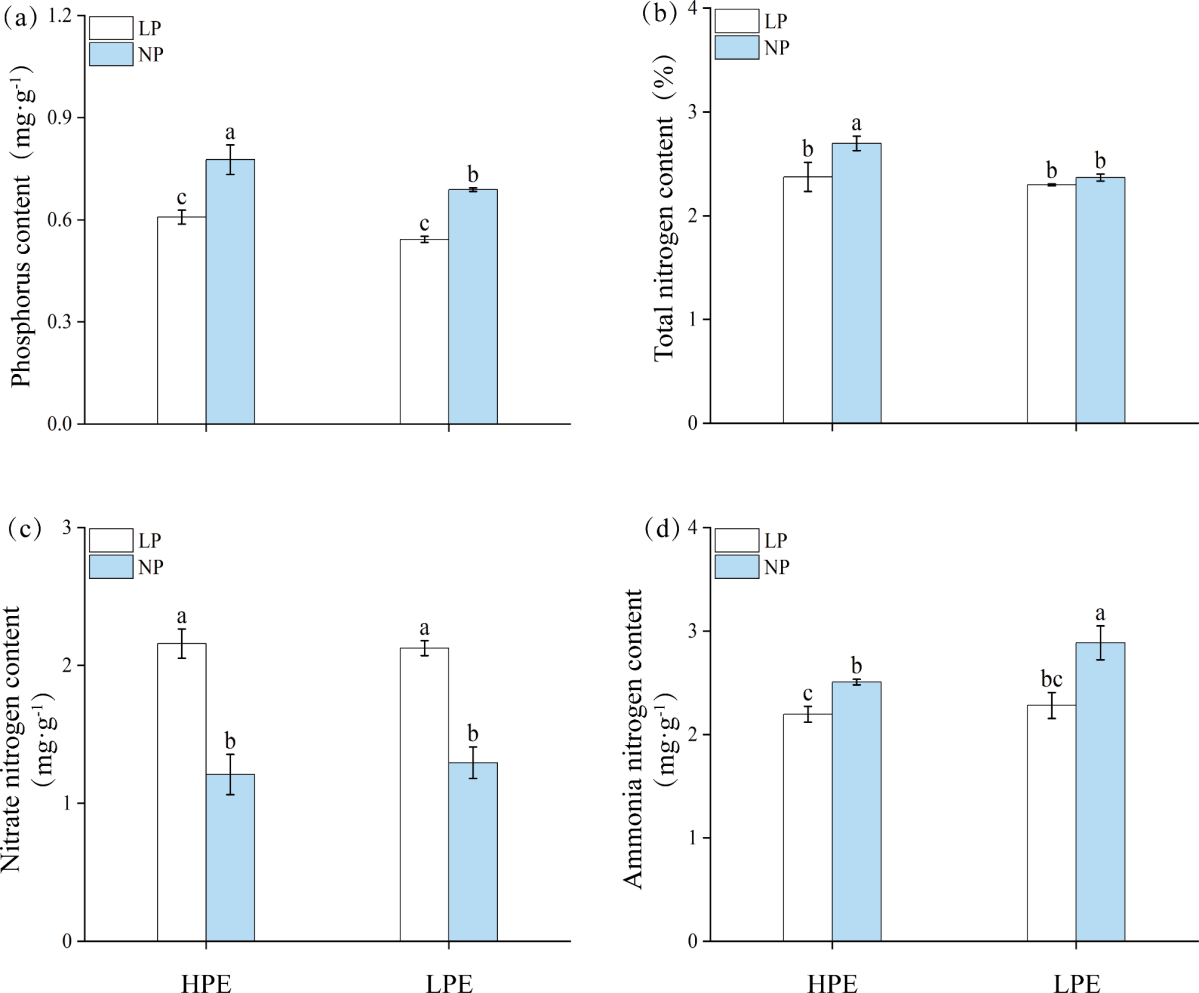
Comparison of contents of phosphorus, nitrogen, nitrate nitrogen and ammonia nitrogen in roots of soybean varieties with different phosphorus efficiencies. (**a**) Phosphorus content. (**b**) Total nitrogen content. (**c**) Nitrate nitrogen content. (**d**) Ammonia nitrogen content. Columns represent the means ± SDs for three biological replicates. Treatments with the same letter are not significantly different from each other at a p-value level of 0.05.

In NP treatment, the contents of phosphorus and total nitrogen in HPE roots were significantly higher than those of LPE, whereas LPE showed higher ammonia nitrogen content than HPE. The difference between HPE and LPE in nitrate nitrogen content was insignificant (Fig. 4). Interestingly, the contents of all four examined major nutrient elements in HPE and LPE were statistically similar under LP treatment (Fig. 4). Compared to NP, contents of phosphorus and ammonia nitrogen were significantly lower under LP treatment in both HPE and LPE varieties (Fig. 4a, d), however the LP nitrate nitrogen contents were significantly increased by 78.51% and 65.12% for HPE and LPE respectively (Fig. 4c). Furthermore, LP stress caused a slight but significant decrease in the nitrogen content in HPE but not in LPE variety (Fig. 4b).

### Activity of key enzymes of the nitrogen metabolism pathway

**Fig. 5:**
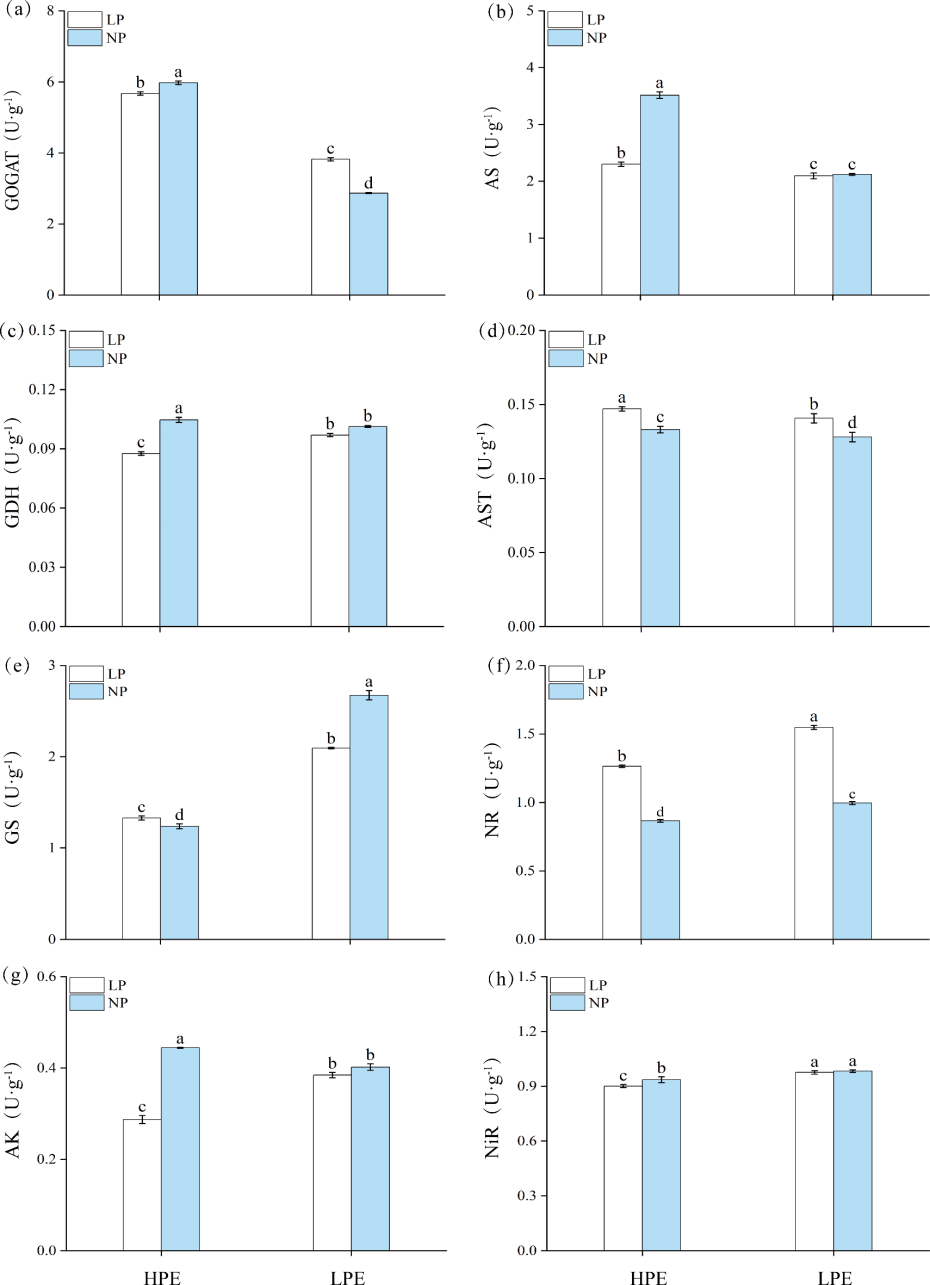
Changes in the activities of key enzymes in the nitrogen metabolism pathway in soybean roots. (**a**) GOGAT activity. (**b**) AS activity. (**c**) GDH activity. (**d**) AST activity. (**e**) GS activity. (**f**) NR activity. (**g**) AK activity. (**h**) NiR activity. Columns represent the means ± SDs for three biological replicates. Treatments with the same letter are not significantly different from each other at a p-value level of 0.05.

In NP treatment, GOGAT, AS, GDH, AST, and AK activities in HPE were significantly higher than in LPE (Fig. 5a-d, g) while HPE activities of GS, NR, and NiR were significantly lower (Fig. 5e, f, h). In LP treatment, GOGAT, AS and AST activities were still significantly higher in HPE roots than in LPE (Fig. 5a, b, d), but GDH, GS, NR, AK and NiR activities were significantly lower than in LPE (Fig. 5c, e-h). Therefore, HPE variety always exhibited higher GOGAT, AS and AST activities and lower GS, NR, and NiR activities than LPE variety under both NP and LP treatments. On the other hand, GDH and AK were the only key enzymes in HPE which were decreased belowed LPE level by the stimulation of LP stress. In HPE variety, the activities of GOGAT, AS, GDH, AK and NiR were significantly decreased under LP treatment compared to NP treatment (Fig. 5a-c, g, h), while the activities of other examined were significantly increased (Fig. 5d-f). In LPE, the activities of GOGAT, AST and NR were significantly increased by 33.10%, 7.69% and 55.00%, respectively (Fig. 5a, d, f) but GS activity was significantly decreased by 21.72% (Fig. 5e) under LP stress compared to NP treatment. No significant change was found in activities of all other four tested enzymes including AS, GDH, AK and NiR. These results showed that LPE could maintain relatively stable activities of nitrogen metabolism pathway enzymes under the LP treatment, while same enzymes in HPE were prone to be inhibited by LP stress.

## Discussion

The content of effective phosphorus in soil is a major determinant of plant growth and productivity^22^. Soybeans have a high demand for phosphorus throughout its life cycle. Phosphorus deficiency is first sensed locally by the root system^23^, and low phosphorus stress can greatly affect root conformation^24^. One of the ways in which plants improve the uptake of phosphorus from the soil is by remodeling the root structure to form an adapted root system^25^. Previous studies have shown that the soybean phosphorus efficiency is closely related to the strength of phosphorus absorption capability in the root system^5^. In this experiment, the root morphology of the two tested soybean varieties exhibited obvious phosphorus deficient symptoms, but the growth of HPE root system (number of lateral roots, root surface area, and root length) was superior to that of LPE under the LP stress (Fig. 1). The LP stress promoted lateral root development and root hair elongation^26^, and the increase in root surface area should facilitate better nutrient uptake from the soil^27^. Previously, it was claimed that phosphorus-efficient varieties improved phosphorus uptake by increasing the number and size of root system, thereby ameliorating the adverse effects of the LP stress on crop growth^7^. The root surface area and number of lateral roots of our HPE variety were significantly higher than those of LPE variety in both NP and LP treatments (Fig. 1b, c), in addition the root length in HPE variety was significantly higher than in LPE variety under the LP stress (Fig. 1d). These results suggested that soybean variety with high phosphorus utilization efficiency could remodel its root structure, increase the contact area between the root system and the soil, and improve the uptake of phosphorus from the soil thereby better maintaining the metabolic balance of phosphorus in the plant body under the LP stress.

After the reduction of nitrate to nitrite, nitrie is eventually converted to glutamate and other amino acids^28^. Common precursors of lysine (Lys), methionine (Met), and threonine (Thr) are produced by a complex pathway from aspartic acid (Asp), which are the main substrates for the synthesis of structural proteins and enzymes required for optimal plant development^29^. In this experiment, the Asp content was significantly reduced in HPE variety under LP stress to a lower level than in LPE (Figs. 2b and 3b), whereas free Thr and Met contents as well as hydrolyzed Lys contents were significantly increased (Figs. 2e and 3c and d). These findings suggested that HPE variety maintained its amino acid metabolic homeostasis by decomposing Asp and increasing the contents of other kinds of amino acids, which in turn improved its adapt ability in the LP stress. Met is known to be the critical limiting amino acid in legumes^30^, and one of its possible mechanisms of action is to stimulate the growth and development of the root system, thereby increasing the crop’s ability to absorb water and nutrients^31^. We suspected that the significant increases of total root length and root surface area of HPE and LPE varieties under the LP stress (Fig. 1c, d) might be possitively related to the significant increase in hydrolyzed Met content (Fig. 2d). Hydrolyzed Met could promote nutrient uptake by soybean roots under the LP stress, maintain nutrient balance in the plant, and help improving stress tolerance. The accumulation of amino acids in roots was associated with levels of amino acid metabolism^32^. The total contents of amino acids of LPE variety was significantly higher than that of HPE variety under the LP stress (Figure S1a; S2a), which suggested that the LP stress slowed down amino acid metabolism in LPE variety but HPE variety was not suffered as much and better adapted to the LP stress. Previous studies have suggested that primary nitrogen assimilation of amino acids in roots occurs via the GS-GOGAT pathway^28,33^. In this experiment, the GS-GOGAT cyclealso seemed to be the main nitrogen pathway. In addition, the LP stress could cause huge changes in amino acid contents (Figure S1a; S2a) therefore it was reasonable to suspect that the changes in GS and GOGAT activities directly affected the amino acid contents. Amino acid metabolism is coordinately regulated by physiological and developmental signals. Metabolisms of glutamate and aspartate are tightly regulated and they can be converted to other amino acids through various biochemical processes^34^. In the LP stress, the AK and AS activities in soybean varieties with different phosphorus efficiencies were both reduced (Fig. 5b, g). HPE variety also showed significantly reduced Asp content (Fig. 2b). These indicated that aspartic acid metabolism was more vigorous in HPE variety under the LP stress. Proline is not only an amino acid required for protein synthesis, but also a precursor for glutamate synthesis. The role of free glutamate in regulating lateral root development is considered an adaptation which enhances the ability of plants to compete for localized nitrogen sources^35^. In this experiment, hydrolyzed glutamate content increased under LP stress (Fig. 2a) in both varieties. The accumulation of free amino acids could serve as a storage for nitrogen and carbon and enhance crop tolerance to adversity^36^. Compared to LPE variety, the proline content of HPE variety in the LP stress was significantly higher, which could effectively increase the synthesis of free glutamate and enhance the absorption and utilization of nitrogen so to maintain better balance of nitrogen metabolism.

Nitrogen is a major component of amino acids, proteins, nucleic acids and various metabolites^37^. The GS-GOGAT metabolic pathway is considered to be the main pathway of nitrogen assimilation in higher plants^35^. Plant root uptake of NO_3_^−^ is reduced to NH_4_^+^ by NR and NiR catalyzed reduction, and the excess NH_4_^+^is immediately assimilated to glutamate and glutamine via the GS-GOGAT cycle and the GDH pathway, and then converted to other types of amino acids^38^. Previous studies claimed that the LP stress reduces plant uptake of nitrogen^39^. In this experiment, nitrate nitrogen content and NR activity were significantly higher and ammonia nitrogen content was significantly lower under LP stress (Figs. 4c and d and 5f) in both HPE and LPE varieties suggesting that the soybean root systems maintained nitrogen homeostasis in the body by regulating the operation rate of nitrogen metabolic pathways under the LP stress. GOGAT activity was significantly higher in HPE than in LPE, while GS and GDH activities were significantly lower in HPE variety under the LP stress (Fig. 5a, c, e). LPE variety showed significantly increased GOGAT activity and decreased GS activity under the LP stress compared with NP condition (Fig. 5a, e). Therefore LPE variety possessed a higher GOGAT/GS ratio than HPE. It was hypothesized that the nitrogen transport pathway of phosphorus-inefficient soybean varieties under LP stress was dominated by the GS-GOGAT cycle. In LP stress, the ammonia nitrogen content of HPE and LPE varieties decreased significantly, while at the same time, the glutamate content increased significantly (Fig. 2a). It indicated that soybean timely converted excess ammonia nitrogen into amino acids during LP stress, which helped to maintain the balance between nitrogen metabolism and amino acid conversion. Lastly, previous studies have shown that nitrogen and phosphorus accumulation is positively correlated with root length to certain extent^40^. In this experiment, the total root length of HPE variety was significantly higher than that of LPE variety, and the contents of nitrogen and phosphorus were also slightly higher than those of LPE variety (Figs. 1d and 4a and b). These results indicated that HPE was able to improve the root growth and better maintained the nutrient metabolism balance under the LP stress by increasing the efficiency of nitrogen and phosphorus uptake in the soil. The above results proved that our phosphorus-efficient soybeans were resilient to low phosphorus stress. We expected that the promotion of phosphorus-efficient soybean would be beneficial for improving soybean acreage and production.

## Conclusions

LP stress affected soybean root growth and disrupted the balance of amino acid metabolism and nitrogen metabolism pathways. HPE variety enhanced soybean survival in LP stress by increasing nutrient (phosphorus, nitrogen, and ammonia nitrogen) content and nitrogen metabolism-related enzyme activities. LP stress weakened amino acid metabolic activities. Aspartic acid metabolism is more vigorous in HPE variety and plays a key role in soybean response to LP stress.

## Electronic supplementary material

Below is the link to the electronic supplementary material.

Supplementary Material 1

## Data Availability

Data is provided within the manuscript or supplementary information files.

## Abbreviations

HPE: High phosphorus utilization
LPE: Low phosphorus utilization
P: Phosphorus
NP: Normal phosphorus treatment
LP: Low phosphorus treatment
GOGAT: Glutamate synthase
GS: Glutamine synthetase
AST: Aspartate transaminase
GDH: Glutamate dehydrogenase
NR: Nitrate reductase
AK: Acetate kinase
NiR: Nitrite reductase
AS: Asparagine synthetase
