# Supplementary material for: Root morphology, nitrogen metabolism and amino acid metabolism in soybean under low phosphorus stress
Source: Sci Rep. 2024 Nov 19;14:28583. doi: 10.1038/s41598-024-79876-0 (PMC11577115; doi:10.1038/s41598-024-79876-0)
Supplement: Supplementary file 1 — Supplementary Material 1 [file 41598_2024_79876_MOESM1_ESM.docx]

**Root morphology, nitrogen metabolism and amino acid metabolism in soybean under low phosphorus stress**

**Meiling Liu^†a^, Mingzhe Zhao^†a^, Guang Yang^a^, Mingze Sun^a^, Ahui Yang^a^, Chang Sun^a^, Hongyu Zhao^a^，Xue Ao^a,*^**

^a^ *College of Agronomy, Shenyang Agricultural University, Shenyang 110866, China*

* For correspondence (e-mails [a2009syau@syau.edu.cn](mailto:a2009syau@syau.edu.cn)).

† These authors contributed equally to this work.

**Supplemental Figures**

**Figure S1**


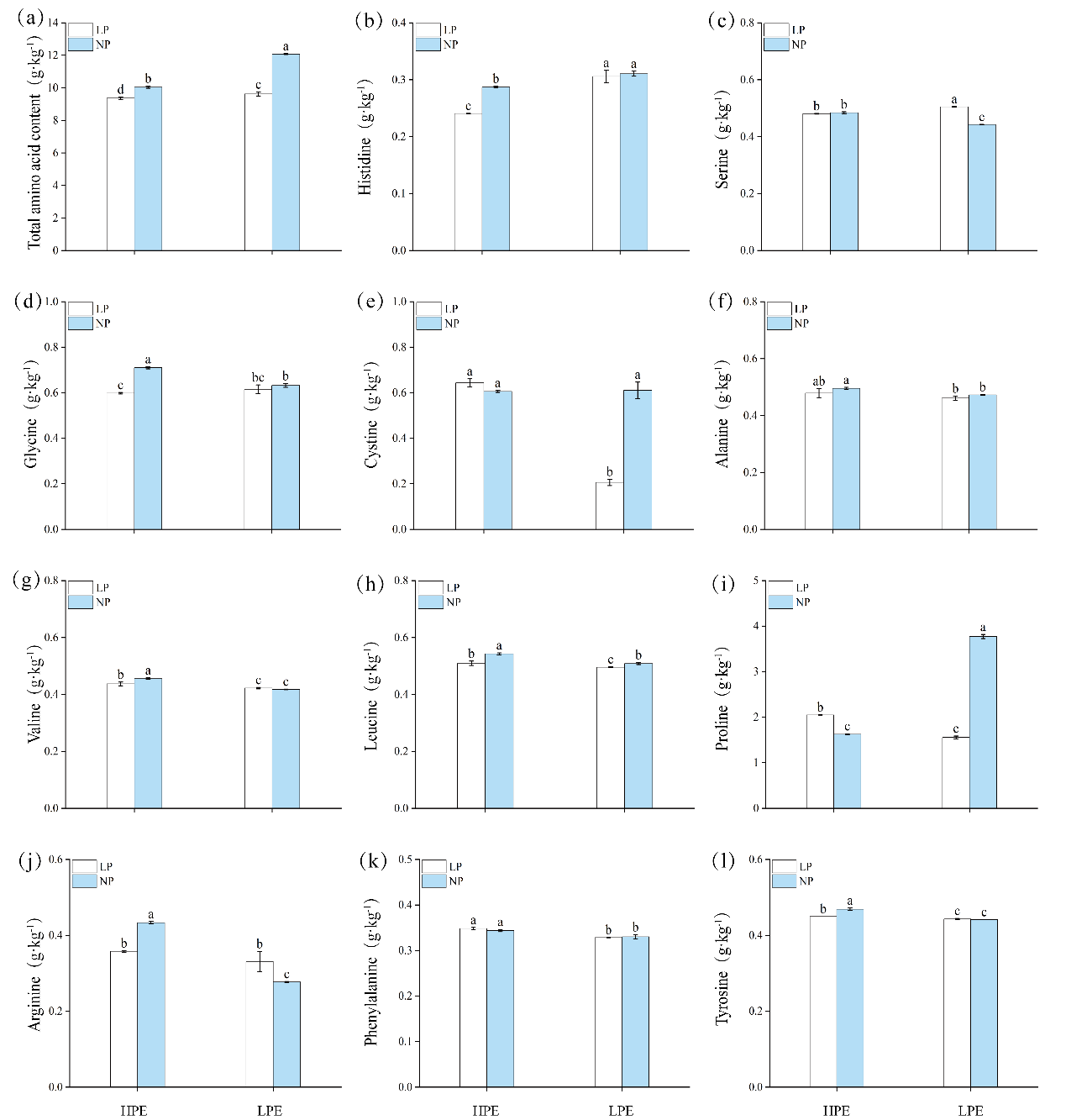


**Figure S1. Changes in total amino acid content and other hydrolyzed amino acid content excluding the six amino acids.** Low Phosphorus Treatment: LP; Normal Phosphorus Treatment: NP; High phosphorus efficiency variety (Liaodou 13) and low phosphorus efficiency variety (Tiefeng 3) are labelled as "HPE" and "LPE" respectively. (**a**) total amino acid content. (**b**) hydrolyzed histidine. (**c**) hydrolyzed serine. (**d**) hydrolyzed glycine. (**e**) hydrolyzed cystine. (**f**) hydrolyzed alanine. (**g**) hydrolyzed valine. (**h**) hydrolyzed leucine. (**i**) hydrolyzed proline. (**j**) hydrolyzed arginine. (**k**) hydrolyzed phenylalanine. (**l**) tyrosine. Columns represent the means ± SDs for three biological replicates. Treatments with the same letter are not significantly different from each other at a p-value level of 0.05.

**Figure S2**


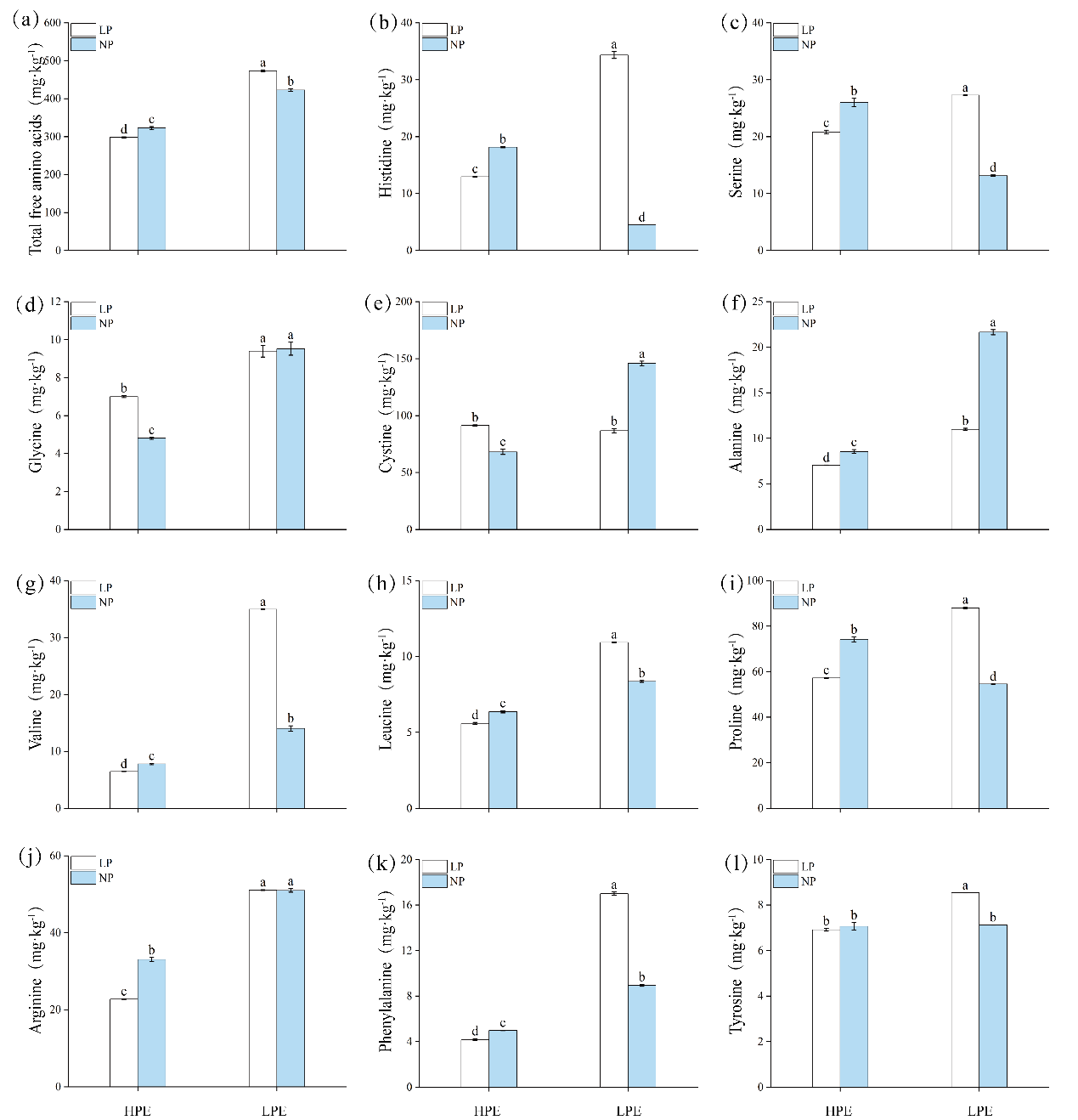


**Figure S2. Changes in total amino acid content and other free amino acid content excluding the six amino acids.** Low Phosphorus Treatment: LP; Normal Phosphorus Treatment: NP; High phosphorus efficiency variety (Liaodou 13) and low phosphorus efficiency variety (Tiefeng 3) are labelled as "HPE" and "LPE" respectively. (**a**) total amino acid content. (**b**) free histidine. (**c**) free serine. (**d**) free glycine. (**e**) free cystine. (**f**) free alanine. (**g**) free valine. (**h**) free leucine. (**i**) free proline. (**j**) free arginine. (**k**) free phenylalanine. (**l**) free tyrosine. Columns represent the means ± SDs for three biological replicates. Treatments with the same letter are not significantly different from each other at a p-value level of 0.05.
